# Platelet-Rich Plasma Prevents In Vitro Transforming Growth Factor-β1-Induced Fibroblast to Myofibroblast Transition: Involvement of Vascular Endothelial Growth Factor (VEGF)-A/VEGF Receptor-1-Mediated Signaling †

**DOI:** 10.3390/cells7090142

**Published:** 2018-09-19

**Authors:** Flaminia Chellini, Alessia Tani, Larissa Vallone, Daniele Nosi, Paola Pavan, Franco Bambi, Sandra Zecchi Orlandini, Chiara Sassoli

**Affiliations:** 1 Department of Experimental and Clinical Medicine, Section of Anatomy and Histology, University of Florence, 50134 Florence, Italy; flaminia.chellini@unifi.it (F.C.); alessia.tani@unifi.it (A.T.); larissa.vallone@unifi.it (L.V.); daniele.nosi@unifi.it (D.N.); sandra.zecchi@unifi.it (S.Z.O.); 2 Transfusion Medicine and Cell Therapy Unit, ”A. Meyer” University Children’s Hospital, 50139 Florence, Italy; paola.pavan@meyer.it (P.P.); franco.bambi@meyer.it (F.B.)

**Keywords:** myofibroblasts, fibrosis, platelet-rich plasma (PRP), vascular endothelial growth factor (VEGF)-A, VEGFR-1/flt-1, Notch-1, transforming growth factor (TGF)-β1/Smad3, α-smooth muscle actin, Connexin 43, confocal immunofluorescence

## Abstract

The antifibrotic potential of platelet-rich plasma (PRP) is controversial. This study examined the effects of PRP on in vitro transforming growth factor (TGF)-β1-induced differentiation of fibroblasts into myofibroblasts, the main drivers of fibrosis, and the involvement of vascular endothelial growth factor (VEGF)-A in mediating PRP-induced responses. The impact of PRP alone on fibroblast differentiation was also assessed. Myofibroblastic phenotype was evaluated by confocal fluorescence microscopy and western blotting analyses of α-smooth muscle actin (sma) and type-1 collagen expression, vinculin-rich focal adhesion clustering, and stress fiber assembly. Notch-1, connexin 43, and VEGF-A expression were also analyzed by RT-PCR. PRP negatively regulated fibroblast-myofibroblast transition via VEGF-A/VEGF receptor (VEGFR)-1-mediated inhibition of TGF-β1/Smad3 signaling. Indeed TGF-β1/PRP co-treated fibroblasts showed a robust attenuation of the myofibroblastic phenotype concomitant with a decrease of Smad3 expression levels. The VEGFR-1 inhibition by KRN633 or blocking antibodies, or VEGF-A neutralization in these cells prevented the PRP-promoted effects. Moreover PRP abrogated the TGF-β1-induced reduction of VEGF-A and VEGFR-1 cell expression. The role of VEGF-A signaling in counteracting myofibroblast generation was confirmed by cell treatment with soluble VEGF-A. PRP as single treatment did not induce fibroblast myodifferentiation. This study provides new insights into cellular and molecular mechanisms underpinning PRP antifibrotic action.

## 1. Introduction

Platelet-rich plasma (PRP) can be defined as a plasma fraction with platelet concentration higher than the baseline concentration in whole blood (approximately 1.5 to 8 times physiological platelet counts). When used with previous platelet activation, it proves to be a cost-effective reservoir of numerous platelet-derived biologically active molecules including growth factors and cytokines, holding a strong potential for improving tissue healing and regeneration [1,2,3,4]. For this reason, it is widely applied in different areas of medicine such as dermatology and aesthetic medicine [5,6,7], plastic surgery [8], dentistry [9,10], musculoskeletal and sport medicine [11,12,13,14], ophthalmology [15,16], gynecology [17], and tissue engineering [18,19]. Its prompt availability from autologous blood, which theoretically disregards concerns of disease transmission or immunogenic reactions, as well as the ease of administration by direct injection in situ not requiring complex equipment or training, represent additional clinical advantages for its use in regenerative medicine protocols [20]. In addition, the excellent safety profile and efficacy, even if in the short term, of allogenic PRP for the treatment of knee osteoarthritis in selected elderly patients or knee involvement in primary Sjögrens syndrome, have been recently demonstrated [21,22], thus opening new perspectives for off-the-shelf PRP therapy for all patients for whom the use of autologous PRP would not be indicated. Many studies have demonstrated that the contribution of PRP to the morpho-functional recovery of various damaged tissues/organs is linkable to its ability to modulate inflammatory responses [23,24,25], promote revascularization [26,27,28], and stimulate the endogenous mechanisms of tissue repair/regeneration by influencing the fate of local cells [29,30,31,32]. The ability of PRP to limit fibrosis in different damaged and/or diseased organs including skeletal muscle [27,33,34,35], myocardium [36], tendon [37], kidney [38], urethra [39], liver [40], skin [41], cornea and conjunctiva [42], and endometrium [43], has also been observed. Fibrosis represents a pathological condition frequently occurring as aberrant response to an injury or chronic diseases at multiple organs. It presents as an excessive tissue scarring due to an overproduction and deposition of extracellular matrix (ECM) mainly attributable to the imbalance between synthesis and degradation of ECM components, particularly collagens, often in association with uncoordinated detrimental contractures. This process may compromise the functionality of resident tissue progenitor cells hampering tissue regeneration, and ultimately leading to destruction of organ architecture and impairment of organ function [44,45]. However, the antifibrotic potential of PRP is still controversial. Indeed some reports show limited effectiveness or inefficacy of this blood-derived product in counteracting the fibrotic response [46,47,48,49], or even a fibrosis development after PRP treatment [35,50,51,52].

Based on these premises, further investigations on the effects of PRP on tissue fibrosis modulation are needed and strong supportive evidence must be provided prior to its clinical use as a new treatment option for fibrosis. Moreover, the bioactive factors contained in PRP actually mediating PRP effects as well as their cellular targets and molecular mechanisms of action need to be clearly identified.

The present study was designed to examine the effects of PRP on the in vitro transition of fibroblastic cells into myofibroblasts, the main drivers of fibrosis [53,54], induced by the profibrotic agent transforming growth factor (TGF)-β1 [45], and to investigate the underlying molecular mechanisms. In addition, given that PRP may contain different profibrotic factors including TGF-β1 [1,2,3,4] and the reported controversial effects of PRP on fibrosis, the impact of PRP alone on fibroblast-myofibroblast differentiation was also evaluated. We found that PRP counteracted myofibroblast generation by interfering with the intracellular signaling mediated by TGF-β1, possibly via activation of vascular endothelial growth factor (VEGF)-A/VEGF receptor (VEGFR)-1 mediated signaling. PRP as single treatment did not promote fibroblast myodifferentiation.

## 2. Materials and Methods

### 2.1. Platelet-Rich Plasma (PRP) Preparation

PRP was obtained from four adult healthy donors, (one female, three males aged 42–54), undergoing a plasma-platelet apheresis (Haemonetics MCS^®^, Haemonetics, Milan, Italy), after receiving proper informed consent. Donors were screened for the main blood-transmitted pathogens, according to current laws on blood transfusion. After collection, the platelet units were stored in a specific shaker incubator, whereas the plasma units were immediately frozen at −80 °C and subsequently thawed at 4 °C for 16 h to obtain the cryoprecipitate by syphoning. Once the cryoprecipitate was obtained, platelets were resuspended in cryoprecipitate and adjusted to a final concentration of 2 × 10^6^/µL. The PRP samples, after being tested for microbiological contamination, were aliquoted and immediately frozen at −80 °C. Platelets’ activation was induced by the addition of a calcium digluconate solution (10%). For the experiments, PRP was diluted with serum-free Dulbecco’s Modified Eagle’s Medium (DMEM; Sigma, Milan, Italy) containing 4.5 g/L glucose or with fibroblast differentiation medium in order to obtain the dilution of 1:50 [32]. PRP was provided in ready-to-use aliquots classified as not suitable for transfusion-infusion purposes. Its use in experimental in vitro protocols does not require Ethical Committee’s approval.

### 2.2. Cell Culture and Treatments

Murine fibroblastic NIH/3T3 cells obtained from American Type Culture Collection (ATCC, Manassas, VA, USA), and human dermal HDFα fibroblast cells from ScienCell (Carlsbad, CA, USA) were grown in proliferation medium (PM) containing DMEM supplemented with 10% fetal bovine serum (FBS) and 1% penicillin/streptomycin (Sigma) at 37 °C in a humidified atmosphere of 5% CO_2_. The cells were induced to differentiate into myofibroblasts by culturing for 48 h and 5 days in differentiation medium (DM) containing DMEM supplemented with 2% FBS and 2 ng/mL TGF-β1 (PeproTech, Inc., Rocky Hill, NJ, USA) as previously reported [55]. In parallel experiments, the cells were cultured in DM or in serum-free DMEM in the presence of PRP at dilution of 1:50 [32] for 48 h and 5 days. In order to evaluate the involvement of VEGF-A/VEGFR-1 mediated signaling in cell responses, some experiments were performed adding to the culture medium a specific ATP competitive inhibitor of VEGF receptor tyrosine kinase activity, KRN633 (IC50  =  170 nM, Santa Cruz Biotechnology, Santa Cruz, CA, USA), rabbit polyclonal anti-VEGFR-1/fms-like tyrosine kinase (Flt-1) neutralizing antibodies (8 µg/mL, Santa Cruz Biotechnology) [56], mouse monoclonal anti-VEGF-A neutralizing antibodies (10 µg/mL, Sigma) [57], or soluble VEGF-A (2 ng/mL and 20 ng/mL, Sigma) [58]. The specificity of the effects of the blocking antibodies was verified by using irrelevant isotype-matched IgG.

### 2.3. Confocal Immunofluorescence

The cells grown on glass coverslips were fixed with 0.5% paraformaldehyde in PBS for 10 min at room temperature. After permeabilization with cold acetone for 3 min and block with 0.5% bovine serum albumin (BSA, Sigma) and 3% glycerol in PBS for 20 min, fixed cells were incubated overnight at 4° C with the following antibodies: mouse monoclonal anti-vinculin (1:100; Sigma); mouse monoclonal anti-α-smooth muscle actin (sma) (1:100, Abcam, Cambridge, UK), rabbit polyclonal anti-type-1 collagen (1:50, Santa Cruz Biotechnology), rabbit polyclonal anti-VEGFR-1/flt-1 (1:50, Santa Cruz Biotechnology), goat polyclonal anti-VEGFR-2/flk-1 (1:10, Santa Cruz Biotechnology), mouse monoclonal anti-VEGFR-3/flt-4 (1:100, Santa Cruz Biotechnology), or mouse monoclonal anti-VEGF-A (1:80, Santa Cruz Biotechnology). The immunoreactions were revealed by incubation with specific anti-rabbit/anti-mouse/anti-goat, Alexa Fluor 488- or 568-conjugated IgG (1:200; Molecular Probes, Eugene, OR, USA) for 1 h at room temperature. In some experiments, the cells were stained with Alexa Fluor 488-labeled phalloidin (1:40; Molecular Probes) to detect actin filament organization (F-actin). In other experiments, counterstaining was performed with propidium iodide (1:30; Molecular Probes) to reveal nuclei. Mouse C2C12 myoblasts expressing VEGFR-2/flk-1 [58] and MCF7 cells expressing VEGFR-3/flt-4 [59] were used as positive controls (data not shown). Negative controls were carried out by replacing the primary antibodies with non-immune serum; cross-reactivity of the secondary antibodies was tested in control experiments in which primary antibodies were omitted. After washing, the coverslips containing the immunolabeled cells were mounted with an antifade mounting medium (Biomeda Gel mount, Electron Microscopy Sciences, Foster City, CA, USA) and observed under a confocal Leica TCS SP5 microscope equipped with a HeNe/Ar laser source for fluorescence measurements and differential interference contrast (DIC) optics (Leica Microsystems, Mannheim, Germany). Observations were performed using a Leica Plan Apo 63×/1.43NA oil immersion objective. Series of optical sections (1024 × 1024 pixels each; pixel size 204.3 nm) 0.4 μm in thickness were taken throughout the depth of the cells at intervals of 0.4 μm. Images were then projected onto a single ‘extended focus’ image. Densitometric analyses of the intensity of vinculin, α-sma, type-1 collagen, and VEGF-A fluorescent signals were performed on digitized images using ImageJ 1.49v software (http://rsbweb.nih.gov/ij) in 20 regions of interest (ROI) of 100 μm^2^ for each confocal stack (at least 10).

### 2.4. Western Blotting

Proteins were extracted from the cells and quantified as reported previously [32]. Forty micrograms of total proteins were electrophoresed on NuPAGE^®^ 4–12% Bis-Tris Gel (Invitrogen, Life Technologies, Grand Island, NY, USA; 200 V, 40 min) and blotted onto polyvinylidene difluoride (PVDF) membranes (Invitrogen, Life Technologies; 30 V, 1 h). The membranes after incubation with Blocking Solution included in the Western Breeze^®^ Chromogenic Western Blot Immunodetection Kit (Invitrogen, Life Technologies) for 30 min at room temperature on a rotary shaker, were incubated overnight at 4 °C with the following antibodies, mouse monoclonal anti-α-sma (1:1000; Abcam), rabbit polyclonal anti-VEGFR-1/flt-1 (1:500, Santa Cruz Biotechnology), and rabbit polyclonal anti-Smad3 (1:1000; Cell Signaling Technology, Danvers, MA, USA). Immunodetection was performed as described in the Western Breeze^®^ Chromogenic Immunodetection kit protocol. After that, the same membranes were washed and immunodetected for the expression of α-tubulin or β-actin assumed as control invariant proteins, by using rabbit polyclonal anti-α-tubulin (1:1000; Merck, Milan, Italy) or mouse monoclonal anti-β-actin antibodies (1:10,000; Sigma).

Densitometric analysis of the bands was performed using ImageJ 1.49v software (http://rsbweb.nih.gov/ij) and the values normalized to control. For a better visualization of the bands a brightness/contrast filter was applied to the original blot images. Representative original blots are shown in Supplementary Materials (Figures S1–S5).

### 2.5. Reverse Transcription Polymerase Chain Reaction (RT-PCR)

The expression levels of Notch-1, Connexin (Cx) 43, and VEGF-A mRNA in NIH/3T3 cells in different culture conditions were determined by RT-PCR. Briefly, total RNA was isolated by extraction with TRIzol Reagent (Invitrogen, Life Technologies), according to the manufacturer’s instructions. One microgram of total RNA was reverse transcribed and amplified with SuperScript One-Step RT-PCR System (Invitrogen, Life Technologies). After cDNA synthesis at 55 °C for 30 min, the samples were pre-denatured at 94 °C for 2 min and then subjected to 40 cycles of PCR performed at 94 °C for 15 s, alternating with 55 °C for 30 s (Notch-1, Cx43, VEGF-A) or 57 °C for 30 s (β-actin, internal control) and 72 °C for 1 min; the final extension step was performed at 72 °C for 5 min. The following mouse gene-specific primers were used. Notch-1 (NM_008714.3), forward 5′-TCGTGCTCCTGTTCTTTGTG-3′ and reverse 5′-TGGTCTCCAGGTCTTCGTCT-3′; Cx43 (X61576.1), forward 5′-AACAGTCTGCCTTTCGCTGT-3′ and reverse 5′-ATCTTCACCTTGCCGTGTTC-3’; VEGF-A (M95200.1) forward 5′-CAGGCTGCTGTAACGATGAA-3’ and reverse 5’-TTTCTCCGCTCTGAACAAGG-3’; β-actin, (NM_007393), forward 5′-ACTGGGACGACATGGAGAAG-3’ and reverse 5’-ACCAGAGGCATACAGGGACA-3’. β-actin mRNA was used as internal standard. Blank controls, consisting of no template (water), were performed in each run. PCR products were separated by electrophoresis on a 1.8% agarose gel and the ethidium bromide-stained bands were quantified by densitometric analysis by using ImageJ 1.49v software (http://rsbweb.nih.gov/ij). β-actin normalization was performed for each result.

### 2.6. VEGF-A Enzyme-Linked ImmunoSorbent Assay (ELISA)

The concentration of VEGF-A in 1:50 diluted PRP samples was measured by the commercial colorimetric sandwich ELISA, Human VEGF assay Kit-IBL (Tecan group Ltd., Männedorf, Switzerland) according to the manufacturer’s recommendations. All standards and samples were analyzed in triplicate. The read of absorbance at 450 nm was performed by using a multiwell scanning spectrophotometer (ELISA reader; Amersham, Pharmacia Biotech, Cambridge, UK).

### 2.7. Statistical Analysis

Data was presented as mean ± standard error of the mean (S.E.M.) as results of at least three independent experiments performed in triplicate. Statistical analysis of differences between the experimental groups was performed using one-way ANOVA with post-hoc Tukey HSD. Significant difference was defined as *p* < 0.05. Calculations were performed using the GraphPad Prism 4.0 statistical software (GraphPad, San Diego, CA, USA).

## 3. Results

### 3.1. PRP Inhibits Fibroblast to Myofibroblast Transition Promoted by TGF-β1

**Figure 1:**
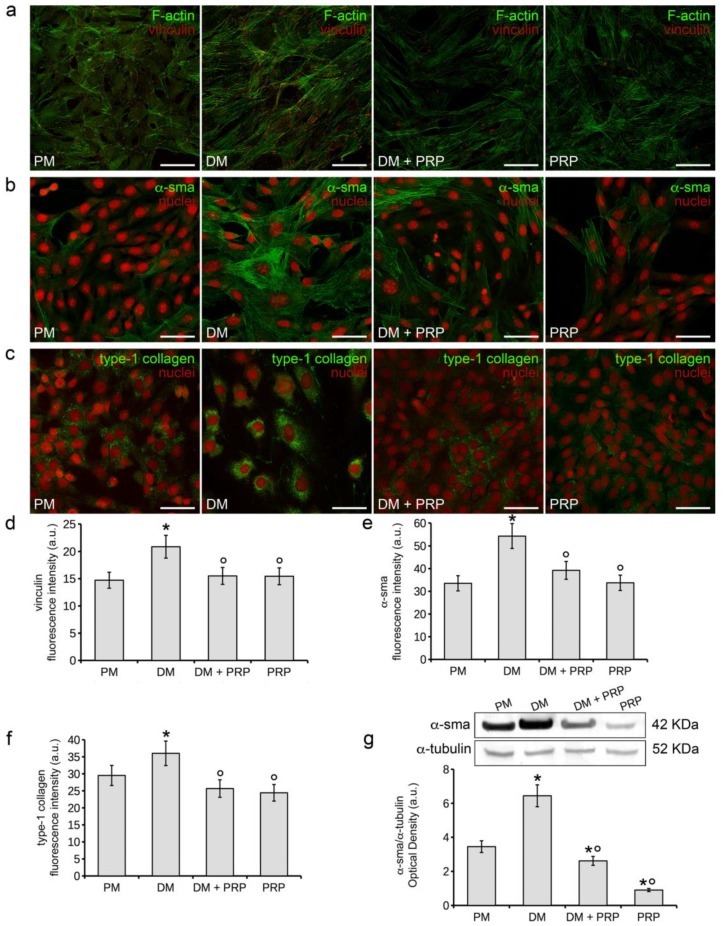
Evaluation of murine NIH/3T3 fibroblast to myofibroblast transition. The cells were induced to differentiate towards myofibroblasts by culturing for 48 h or 5 days in differentiation medium (DM, low serum medium plus 2 ng/mL TGF-β1). Cells cultured in proliferation medium (PM) served as control, undifferentiated cells. To evaluate the effects of PRP on TGF-β1-induced fibroblast-myofibroblast transition, cells were cultured in DM added with 1:50 diluted PRP (DM + PRP). In addition, the cells were cultured in the presence of 1:50 serum-free medium diluted PRP (PRP). (**a**–**c**) Representative confocal fluorescence images of the cells (**a**) stained with Alexa Fluor 488-conjugated phalloidin to reveal F-actin and immunostained with antibodies against vinculin, (**b**) immunostained with antibodies against α-sma or (**c**) type-1 collagen. In (**b**,**c**), nuclei are counterstained with propidium iodide. Scale bar: 50 µm. (**d**–**f**) Histograms showing the densitometric analyses of the intensity of the fluorescence signals for each marker, performed on digitized images. (**g**) Western blotting analysis of α-sma expression. Histogram shows the densitometric analysis of the bands normalized to α-tubulin. Data shown are mean ± S.E.M. and represent the results of at least three independent experiments performed in triplicate. Significance of difference: * *p* < 0.05 vs. PM; ° *p* < 0.05 vs. DM.

In order to promote fibroblast differentiation towards myofibroblasts, murine NIH/3T3 and human HDFα fibroblastic cells were cultured in differentiation medium (DM) consisting of a low serum medium (DMEM plus 2% FBS) with the addition of the profibrotic agent TGF-β1 (2 ng/mL) for 48 h and 5 days [55]. Cells cultured in proliferation medium (PM, DMEM plus 10% FBS) served as control, undifferentiated cells. To evaluate the effects of PRP on such TGF-β-induced cellular process, PRP was added to the DM (1:50 dilution, DM + PRP). In addition, the effects of PRP alone on fibroblast-myofibroblast differentiation were evaluated by culturing the cells in the presence of PRP diluted in serum-free medium (1:50) for different times as above. Confocal immunofluorescence analysis revealed that after 48 h of culture, TGF-β1 induced a prominent cytoskeletal rearrangement in NIH/3T3 cells as compared to control cells, with the formation of massive well-defined actin in parallel-arranged stress fibers and of vinculin rich-focal adhesion sites mainly located at the distal ends of the stress fibers (Figure 1a,d). These effects were associated with an increase in both the expression of α-sma (48 h) (Figure 1b,e), a well-known myofibroblastic marker, which appeared mainly localized along the stress fiber course, and of type-1 collagen (5 days), which was distributed throughout the cytoplasm (Figure 1c,f). The TGF-β1-induced increase of α-sma expression was confirmed by western blotting analysis (Figure 1g). PRP was able to strongly reduce the phenotypical changes induced by TGF-β1; indeed TGF-β1-stimulated cells in the presence of PRP (DM + PRP) exhibited a marked reduction of both stress fiber assembly and redistribution of vinculin to focal adhesion sites (Figure 1a,d) and a downregulation of α-sma (Figure 1b,e,g) and type-1 collagen (Figure 1c,f) expression. Notably, PRP as a single treatment did not significantly modify the morphological pattern of fibroblasts, whose cytoskeletal apparatus appeared comparable to that of the control cells (Figure 1a,d) as well as the expression levels of α-sma (Figure 1b,e,g) and type-1 collagen (Figure 1c,f), which appeared similar or even lower than those of controls.

**Figure 2:**
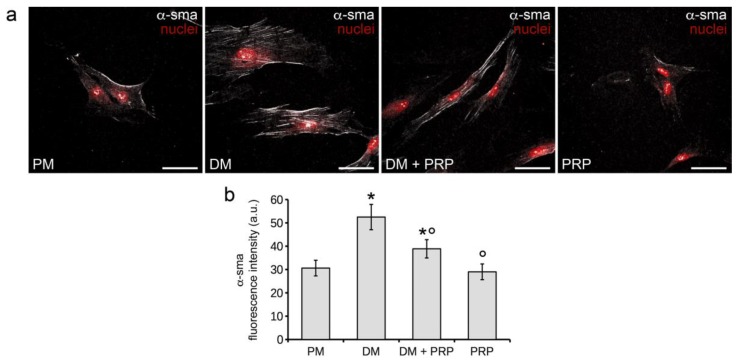
Evaluation of human HDFα fibroblast to myofibroblast transition. The cells were induced to differentiate towards myofibroblasts by culturing for 48 h in differentiation medium (DM, low serum medium plus 2 ng/mL TGF-β1). Cells cultured in proliferation medium (PM) served as control undifferentiated cells. To evaluate the effects of PRP on TGF-β1-induced fibroblast-myofibroblast transition, cells were cultured in DM added with 1:50 diluted PRP (DM + PRP). In addition, the cells were cultured in the presence of 1:50 serum-free medium diluted PRP (PRP). (**a**) Representative confocal fluorescence images of the cells immunostained with antibodies against α-sma and counterstained with propidium iodide to label nuclei. Scale bar: 50 µm. (**b**) Histogram showing the densitometric analyses of the intensity of α-sma fluorescence signal performed on digitized images. Data shown are mean ± S.E.M. and represent the results of at least three independent experiments performed in triplicate. Significance of difference: * *p* < 0.05 vs. PM; ° *p* < 0.05 vs. DM.

The capability of PRP to inhibit TGF-β1-induced myofibroblast differentiation or to prevent differentiation when used as a single treatment was confirmed on human HDFα fibroblasts (Figure 2). Indeed, when these cells were cultured in DM + PRP they appeared spindle shaped (Figure 2a) and showed a reduced expression and organization of α-sma along the stress fibers, with respect to the differentiating cells cultured in DM (Figure 2a,b). The cells cultured with PRP alone appeared superimposable to control cells (PM) exhibiting a similar morphology and low α-sma expression levels.

**Figure 3:**
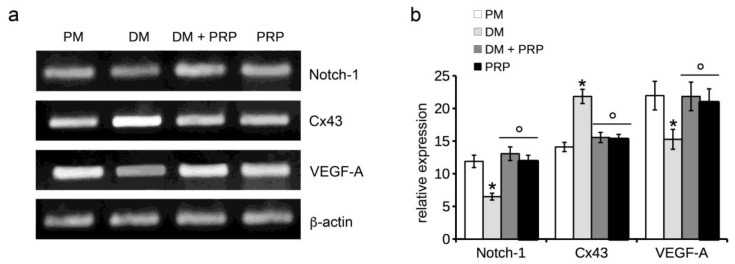
RT-PCR analysis of Notch-1, Connexin 43 (Cx43), and VEGF-A expression. NIH/3T3 fibroblastic cells were cultured in differentiation medium (DM, low serum medium plus 2 ng/mL TGF-β1) in the absence or presence of 1:50 diluted PRP (DM + PRP) or in the presence of 1:50 serum-free medium diluted PRP (PRP). Cells cultured in proliferation medium (PM) served as controls, undifferentiated cells. (**a**) Representative agarose gels. (**b**) Histogram showing the densitometric analyses of the bands normalized to β-actin. Data shown are mean ± S.E.M. and represent the results of at least three independent experiments performed in triplicate. Significance of difference: * *p* < 0.05 vs. PM; ° *p* < 0.05 vs. DM.

Fibroblast myodifferentiation was also assessed by the evaluation of Notch-1 and Connexin (Cx) 43 mRNA expression by RT-PCR analysis, based on previous studies demonstrating that Notch-1 signaling and Cx43 contribute negatively and positively regulate fibroblast-myofibroblast transition, respectively [60,61,62]. A statistically significant downregulation of Notch-1 mRNA expression was observed in NIH/3T3 fibroblasts cultured in DM compared to control cells according to our previous observations [60]; this event was prevented by treatment with PRP (Figure 3). Conversely, TGF-β1 induced an upregulation of Cx43 mRNA expression that was abrogated by PRP (Figure 3). No variation in the expression of these genes was detected in the cells cultured with PRP alone, as compared to control cells (Figure 3).

### 3.2. PRP Prevents Fibroblast-Myofibroblast Transition via VEGF-A/VEGFR-1-Mediated Inhibition of TGF-β1/Smad3 Signaling

**Figure 4:**
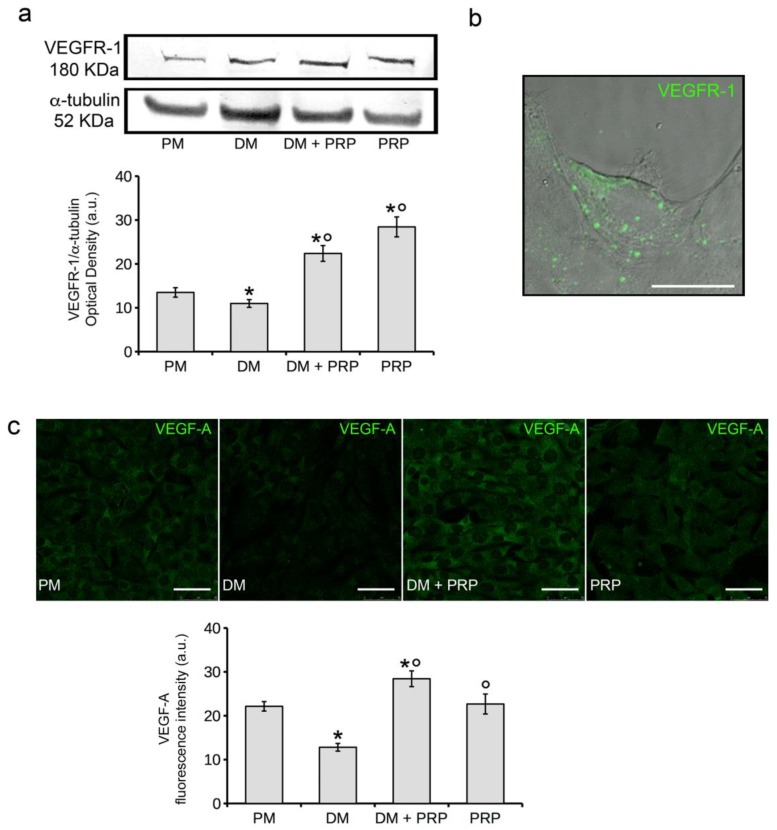
Fibroblast VEGFR-1 and VEGF-A expression. NIH/3T3 fibroblastic cells were cultured in differentiation medium (DM, low serum medium plus 2 ng/mL TGF-β1) in the absence or presence of 1:50 diluted PRP (DM + PRP) or in the presence of 1:50 serum-free medium diluted PRP (PRP). Cells cultured in proliferation medium (PM) served as control undifferentiated cells. (**a**) Western blotting analysis of VEGFR-1 expression. Histogram shows the densitometric analysis of the bands normalized to α-tubulin. (**b**) Representative superimposed differential interference contrast (DIC) and confocal fluorescence images of control cells immunostained with antibodies against VEGFR-1 showing the cellular localization of VEGFR-1; the staining (green) is mainly localized at the cell surface. Scale bar: 20 µm. (**c**) Representative confocal fluorescence images of the cells immunostained with antibodies against VEGF-A. Scale bar: 50 µm. Histogram shows the densitometric analysis of the intensity of VEGF-A fluorescence signal performed on digitized images. Data shown are mean ± S.E.M. and represent the results of at least three independent experiments performed in triplicate. Significance of difference: * *p* < 0.05 vs. PM; ° *p* < 0.05 vs. DM.

To investigate the molecular mechanisms by which PRP exerts the inhibitory effect on TGF-β1-induced fibroblast-myofibroblast transition, we next evaluated the involvement of VEGF-A-mediated signaling in our cell system, based on the following considerations: PRP is a source of VEGF-A [63,64] and VEGF-A has been demonstrated to inhibit TGFβ-1-mediated pathway in other cell types [65]. When assessed by a commercially available ELISA Kit, VEGF-A concentration in our PRP samples was 65 ± 3.8 pg/mL. As judged by western blotting (Figure 4a) and confocal immunofluorescence (Figure 4b) analyses, NIH/3T3 cells expressed VEGF Receptor (VEGFR)-1, but not VEGFR-2 or VEGFR-3 (data not shown), in accordance to previous report investigating VEGFRs expression in lung fibroblasts [66].

In particular we found that VEGFR-1 expression was reduced in the cells cultured in DM compared to the controls; the addition of PRP prevented this reduction (Figure 4a). PRP alone induced an increase of VEGFR-1 expression as compared to controls (Figure 4a). Of note, the cells cultured in DM showed a downregulation of mRNA and protein expression levels of VEGF-A as compared to control cells. By contrast, VEGF-A expression in the cells cultured in DM + PRP or with PRP alone was higher or similar to that of control cells (Figure 3 and Figure 4c), respectively, further stressing a critical role of VEGF-A in fibroblast myodifferentiation.

**Figure 5:**
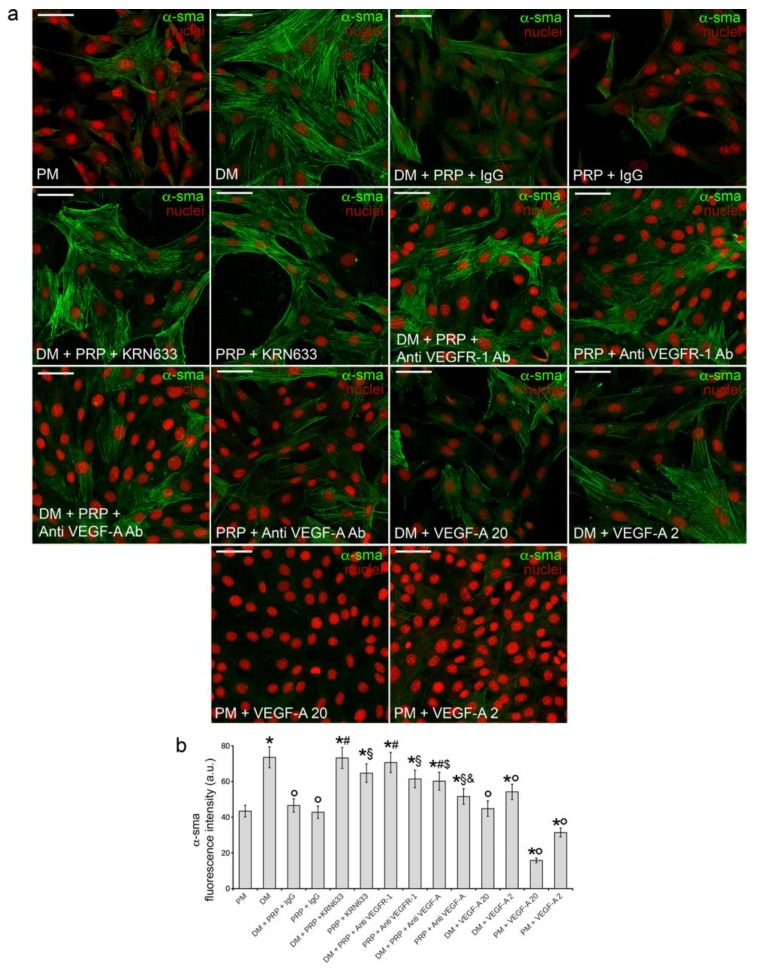
Effect of VEGFR-1 inhibition, VEGF-A neutralization, and stimulation with soluble VEGF-A on α-sma expression: confocal immunofluorescence analysis. NIH/3T3 fibroblastic cells were cultured in differentiation medium (DM, low serum medium plus 2 ng/mL TGF-β1) in the absence or presence of 1:50 diluted PRP + irrelevant IgG (DM + PRP + IgG) or in the presence of 1:50 serum-free medium diluted PRP + IgG. To evaluate the involvement of VEGF-A/VEGFR-1 mediated signaling in PRP-induced fibroblast response, the cells were treated with the selective pharmacological VEGFR inhibitor, KRN633 (DM + PRP + KRN633; PRP + KRN633) or with anti-VEGFR-1 neutralizing antibodies (8 µg/mL; DM + PRP+ Anti VEGFR-1 Ab; PRP + Anti VEGFR-1 Ab) or anti-VEGF-A neutralizing antibodies (10 µg/mL ; DM + PRP+ Anti VEGF-A Ab; PRP + Anti VEGF-A Ab). In parallel experiments the cells were cultured in DM or PM in the presence of two different concentrations of soluble VEGF-A (20 ng/mL, DM + VEGF-A 20, PM + VEGF-A 20; 2 ng/mL, DM + VEGF-A 2, PM + VEGF-A 2). Cells cultured in proliferation medium (PM) served as control undifferentiated cells. (**a**) Representative confocal fluorescence images of the cells immunostained with antibodies against α-sma and counterstained with propidium iodide to reveal nuclei. Scale bar: 50 µm. (**b**) Histogram showing the densitometric analysis of the intensity of α-sma fluorescence signal performed on digitized images. Data shown are mean ± S.E.M. and represent the results of at least three independent experiments performed in triplicate. Significance of difference: * *p* < 0.05 vs. PM; ° *p* < 0.05 vs. DM; ^#^ *p* < 0.05 vs. DM + PRP + IgG; ^§^ *p* < 0.05 vs. PRP + IgG; ^$^ *p* < 0.05 vs. DM + PRP + Anti VEGFR-1 Ab; ^&^ *p* < 0.05 vs. PRP + Anti VEGFR-1 Ab.

**Figure 6:**
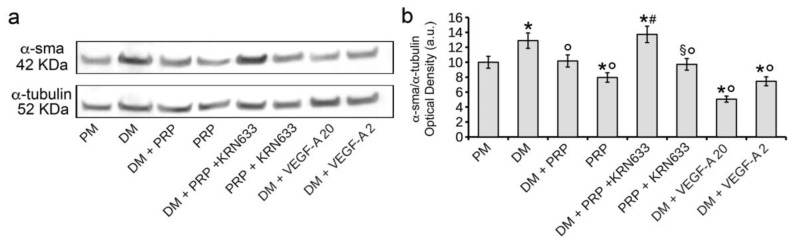
Effect of VEGFR-1 inhibition and of stimulation with soluble VEGF-A on α-sma expression: western blotting analysis. NIH/3T3 fibroblastic cells were cultured in differentiation medium (DM, low serum medium plus 2 ng/mL TGF-β1) in the absence or presence of 1:50 diluted PRP (DM + PRP) or in the presence of 1:50 serum-free medium diluted PRP (PRP). To evaluate the involvement of VEGF-A/VEGFR-mediated signaling in PRP- induced fibroblast response, the cells were treated with the selective pharmacological VEGFR inhibitor, KRN633 (DM + PRP + KRN633; PRP + KRN633). In parallel experiments the cells were cultured in DM in the presence of two different concentrations of soluble VEGF-A (20 ng/mL, DM + VEGF-A 20; 2 ng/mL, DM + VEGF-A 2). Cells cultured in proliferation medium (PM) served as control undifferentiated cells. (**a**) Representative western blots of α-sma and tubulin expression. (**b**) Histogram showing the densitometric analysis of the bands normalized to α-tubulin. (**b**) Data shown are mean ± S.E.M. and represent the results of at least three independent experiments performed in triplicate. Significance of difference: * *p* < 0.05 vs. PM; ° *p* < 0.05 vs. DM; ^#^ *p* < 0.05 vs. DM + PRP; ^§^ *p* < 0.05 vs. PRP.

Interestingly, the treatment of fibroblasts cultured in DM + PRP with the selective pharmacological VEGFR inhibitor, KRN633, or with anti-VEGFR-1 or anti-VEGF-A neutralizing antibodies prevented the effect of downregulation of α-sma expression promoted by PRP (Figure 5 and Figure 6) demonstrating the involvement of VEGF-A/VEGFR-1 signaling in mediating the inhibitory effects of PRP on TGF-β1-stimulated fibroblast-myofibroblast transition. The treatment with KRN633 or with the VEGFR-1 or VEGF-A blocking antibodies also abrogated the inhibitory effects of PRP alone on myofibroblast generation (Figure 5 and Figure 6), further confirming the involvement of VEGF-A/VEGFR-1 underlying PRP action. The role of VEGF-A in fibroblast-myofibroblast differentiation was further assayed in experiments in which the NIH/3T3 cells were exposed to different concentrations (2 ng/mL and 20 ng/mL) of soluble VEGF-A. As expected, the addition of soluble VEGF-A to DM caused a marked decrease of α-sma expression in TGF-β1-treated fibroblasts (Figure 5 and Figure 6); interestingly VEGF-A was also capable to reduce the basal α-sma expression levels as judged by the results of the experiments performed by adding soluble VEGF-A to PM (Figure 5), suggesting a role of VEGF-A in the promotion of α-sma reduction independent or, at least, partially independent from its ability to modulate TGF-β1 signaling.

**Figure 7:**
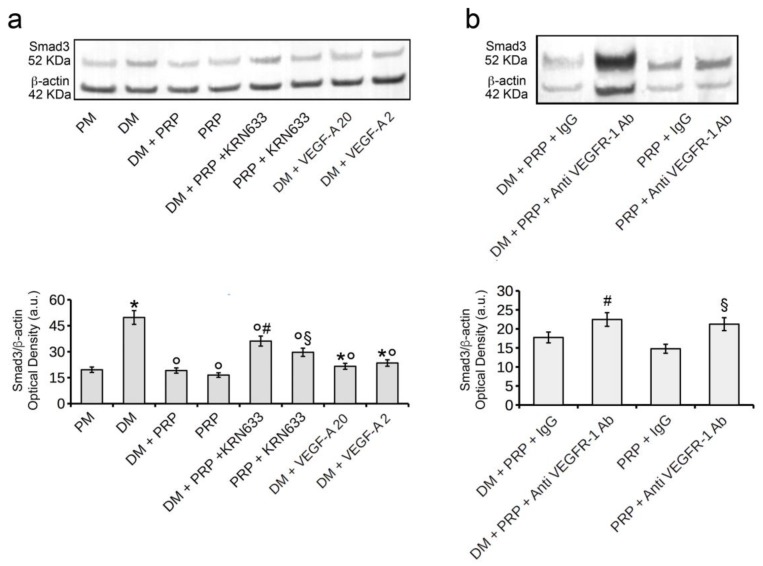
Western blotting analysis of Smad3 expression. (**a**) NIH/3T3 fibroblastic cells were cultured in differentiation medium (DM, low serum medium plus 2 ng/mL TGF-β1) in the absence or presence of 1:50 diluted PRP (DM + PRP) or in the presence of 1:50 serum-free medium diluted PRP (PRP). To evaluate the involvement of VEGF-A/VEGFR-1 mediated signaling in the PRP-induced fibroblast response, the cells were treated with the selective pharmacological VEGFR inhibitor, KRN633 (DM + PRP + KRN633; PRP + KRN633). In parallel experiments the cells were cultured in DM in the presence of two different concentrations of soluble VEGF-A (20 ng/mL, DM + VEGF-A 20; 2 ng/mL, DM + VEGF-A 2). Cells that were cultured in proliferation medium (PM) served as control undifferentiated cells. (**b**) The cells were cultured in DM + PRP or PRP alone in the presence of irrelevant isotype-matched IgG (DM + PRP + IgG; PRP + IgG) or anti-VEGFR-1 neutralizing antibodies (8 µg/mL; DM + PRP + Anti VEGFR-1 Ab; PRP + Anti VEGFR-1 Ab. Representative western blots of Smad3 and β-actin expression are shown. Histograms show the densitometric analysis of the bands normalized to β-actin. Data shown are mean ± S.E.M. and represent the results of at least three independent experiments performed in triplicate. Significance of difference: in (**a**), * *p* < 0.05 vs. PM; ° *p* < 0.05 vs. DM; ^#^ *p* < 0.05 vs. DM + PRP; ^§^ *p* < 0.05 vs. PRP; in (**b**), ^#^ *p* < 0.05 vs. DM + PRP + IgG; ^§^ *p* < 0.05 vs. PRP + IgG.

Finally, the expression of Smad3, the TGF-β1 downstream signaling molecule [45], appeared downregulated in fibroblasts cultured in DM + PRP or DM + VEGF-A compared to cells cultured in DM (Figure 7a). Conversely, the blockade of VEGFR-1 by KRN633 (Figure 7a) or anti VEGFR-1 neutralizing antibodies in the cells cultured in DM + PRP (Figure 7b) induced an increase in Smad3 expression levels compared to those of cells cultured in the absence of the VEGFR-1 inhibitor (DM + PRP or DM + PRP + IgG).

The expression levels of Smad3 in the cells cultured with PRP alone were similar to those of control cells, whereas they increased when the cells were treated with KRN633 (Figure 7a) or with anti-VEGFR-1 neutralizing antibodies (Figure 7b). These findings demonstrated that PRP and VEGF-A/VEGFR-1 signaling counteracted the fibroblast-myofibroblast transition by interfering with the TGF-β1-mediated intracellular pathway.

## 4. Discussion

Myofibroblasts are a population of cells which reside in the ECM of all organs and result from the activation and differentiation of different precursor cells, including resident fibroblasts, essentially promoted by soluble fibrogenic factors such as TGF-β1 and by mechanical signals [45,53,54,67,68]. They are characterized by immunophenotypical and ultrastructural features of both smooth muscle cells and fibroblasts, exhibiting bundles of contractile actin/myosin-containing stress fibers, large focal adhesion complexes, and by the expression of α-sma together with the typical prominent rough endoplasmic reticulum of synthetically active fibroblasts [67]. Myofibroblasts are believed to be the key cell effectors of tissue scarring [53,54]. Scar formation represents a crucial step of the normal physiological healing response to tissue injury in any organ, essentially required to rapidly restore tissue integrity. Myofibroblasts, capable of actively producing abundant ECM proteins and exerting contractile forces, are considered major contributors to the formation and the remodeling of a contractile scar, which enables the size reduction of the wound as well as its closure [69,70]. These cells are only transiently present in normal repair process of acute damage, and then progressively disappear—possibly undergoing apoptosis or reverting to inactive phenotype—once the provisional scar is degraded and tissue regeneration is accomplished. By contrast, myofibroblasts persist in the activated state in fibrotic diseases, characterized by an excessive deposition of dense ECM which, in the worst condition, provokes the disruption of both the physiological organ architecture and function [44,54]. Fibrosis may therefore be considered as a prolonged, exacerbated, and unresolved tissue repair process, occurring in response to repeated or chronic tissue damage, irrespective of the underlying etiology. Eradication of the etiology may result, in some cases, in fibrosis resolution; unfortunately, effective treatment to eliminate the injury cause is not always available. In addition, it must be considered that most human fibrotic diseases are often multifactorial in origin, making it virtually impossible to act on the noxious causes.

The current therapeutic options for fibrosis are of limited efficacy [71,72,73] and, at present, organ transplantation, when possible, represents the only option for patients affected by fibrosis, with all the correlated critical issues and concerns. Thus, since severe fibrosis is estimated to account for up to 45% of deaths in industrialized countries [74], the development of alternative and effective therapies aimed to attenuate fibrotic response or even to induce its regression, represents a major, and still unmet, medical need, with a high impact on health care system. Given that myofibroblasts are the master effectors of fibrosis in most organs, they could represent potential preferential therapeutic targets. In others words, the modulation of differentiation, life-span, and functionality of these fibrogenic cells in developing or mature pathological scars, or even before ECM deposition becomes pathological, may represent effective strategical antifibrotic options. As smartly discussed in recent papers [41,53,73,74,75], several possibilities may exist to accomplish this task, which are not mutually exclusive, including modulation of the “feed-forward” loops which support myofibroblast persistence, induction of myofibroblast dedifferentiation/reversion to an inactive phenotype, or reprogramming or promotion of cell senescence or apoptosis. In such a view, demonstrating the ability of PRP to prevent TGF-β1-induced fibroblast-myofibroblast transition by negatively affecting the canonical profibrotic TGF-β1-Smad3-mediated signaling and not induce per se fibroblast myodifferentiation is of potential clinical interest. Indeed, this suggests that PRP may hold some promise as a tool non-organ-specific capable to reduce the amount of fibrogenic myofibroblasts, prerequisite for sustained fibrosis resolution. Moreover, taking into account data showing the ability of TGF-β1 to confer an apoptosis-resistant phenotype to fibroblasts or myofibroblasts via AKT activation [76,77], PRP, by antagonizing TGF-β1 signaling, may exert its antifibrotic action also by promoting myofibroblast apoptosis.

We have recently demonstrated that PRP alone and, to a greater degree, in combination with bone marrow-derived mesenchymal stromal cells stimulates in vitro proliferation and differentiation of myogenic progenitors including satellite cells, suggesting that it may favor endogenous repair/regeneration mechanisms in damaged skeletal muscle tissue [32]. It is well-known that in cases of severe and extended skeletal muscle damage, the regenerative ability of the tissue may be hampered by the occurrence of a fibrous scar replacing the injured tissue [78]. In such a view, the findings of the present study are very interesting, expanding the list of potential beneficial effects of PRP in supporting skeletal muscle tissue regeneration; indeed, it may contribute to directly activate the resident muscle progenitor cells and, in parallel, modulate myofibroblast generation and functionality reducing the fibrotic response, thus contributing to the recreation of a more hospitable and conductive microenvironment for muscle progenitor functionality. These effects are consistent with the observed positive outcomes achieved after injections of PRP in damaged skeletal muscles [27,33,34,35].

The results of this study are also in accordance with findings of previous reports demonstrating the capability of plasma rich in platelet derived growth factors to inhibit and revert TGF-β1-induced myodifferentiation of conjunctival [79] and gingival fibroblasts, as well as not to stimulate, when administered as single treatment, the myofibroblast phenotype acquisition [80]. By contrast, other studies proved that PRP promotes the myofibroblastic differentiation process [81,82,83,84,85]. These contradictory PRP-elicited biological responses may be attributed to different cell type responsiveness or, more likely, to the heterogeneity of PRP preparation techniques and formulations containing different concentration of interplaying growth factors [64,86,87] exerting, at the same time, profibrotic (such as TGF-β) and antifibrotic actions (such as FGF-2) [88]. In addition, the different PRP dosages used could be determinant when considering the dose-dependence of some fibroblasts’ responses [89,90,91].

This issue highlights and stresses the need for standardization of PRP preparation techniques and application protocols in order to perform meaningful comparative analyses, enable reproducibility, reach reliable conclusions regarding the efficacy of this blood-derivate and, therefore, attain the effective therapeutic translation of this approach.

Of particular interest are the results of the present study demonstrating that PRP acts through VEGF-A/VEGFR-1(Flt-1)-mediated signaling to antagonize TGF-β1/Smad3 signaling and inhibit or prevent myofibroblast generation, thus shedding some light into the molecular mechanisms by which PRP exerts its antifibrotic effect. Indeed, we observed that the inhibition of VEGF-A/VEGFR-1 mediated signaling by KRN633 or by anti-VEGFR-1 neutralizing antibodies or the neutralization of VEGF-A, blocked the effects of PRP on inhibition of the TGF-β1-induced fibroblast-myofibroblast transition, and on downregulation of Smad3 expression. In our cell system, the inhibition of VEGFR-1 seems to elicit more marked effects than VEGF-A neutralization suggesting a potential role of the other VEGFR-1 ligands such as VEGF-B or Placental growth factor (PlGF) in mediating the response of the cells to PRP. The concentration of VEGF-B and PlGF in our PRP samples has yet to be analyzed and further investigations are required to evaluate the impact of these factors in fibroblast-myofibroblast transition. However the role ascribed to PlGF seems to be essentially profibrotic [92,93,94,95].

We also found that PRP prevented the reduction of fibroblast expression of VEGFR-1 as well as of VEGF-A induced by TGF-β1, consistent with previous studies [80]. These results, beside confirming the cross-talk between TGF-β1 and VEGF-A pathways, as observed in other cell types [96,97], and contributing to stress the role of VEGF-A/VEGFR-1 in the negative regulation of fibroblast myodifferentation, may suggest that factors released by PRP could also be able to modulate the responsiveness of fibroblastic cells to VEGF-A.

The VEGF-A/VEGFR-1 pathway may be also involved in mediating the ability of PRP, when used as single treatment, of not promoting per se fibroblast differentiation towards myofibroblasts on the basis of the following results: (i) PRP alone induced a slight increase in VEGFR-1 expression and (ii) the blockade of VEGFR-1 and the neutralization of VEGF-A abrogated the preventive effects of PRP on myofibroblast generation inducing an upregulation of α-sma expression. In addition, our results, showing an increase of Smad3 expression levels in the cells cultured with PRP alone (i.e., in the absence of differentiation medium containing TGF-β1) in the presence of VEGFR-1 inhibitors, suggest that VEGF-A/VEGFR-1 signaling negatively interferes with the signaling mediated by TGF-β1, likely contained in PRP. However, the possibility that VEGF-A released by PRP may affect α-sma expression regardless of modulation of TGF-β1 signaling cannot be excluded. We can speculate that VEGF-A might exert its inhibitory action by cross-talking with other factors contained in PRP capable to act as antifibrotic agents, such as FGF-2 [64,86,87,88], or profibrotic ones such as PDGF [64,86,87,98], as reported in other cell types [99,100].

On the other hand, the role of VEGF-A on negative regulation of TGF-β1-induced fibroblast myodifferentiation, has been further investigated and confirmed by experiments including soluble VEGF-A. Interestingly, the reduction of α-sma expression levels observed in the cells cultured in differentiation medium, and even more in proliferation medium (i.e., in the absence of TGF-β1) and stimulated with soluble VEGF-A, below the levels observed in the cells cultured in proliferation medium alone, supports the potential of a role for VEGF-A in inhibition of myofibroblast differentiation, independent, or at least partially independent, from its ability to modulate TGF-β1 signaling.

Our experimental evidences supporting the involvement of VEGF-A mediated pathway in negatively modulating fibrosis development and its ability to interplay with TGF-β1 signaling are consistent with previous in vitro observations showing that kidney cortex cells stably overexpressing VEGF-A, upon TGF-β1 stimulation, showed a strong reduction of Smad3 expression and phosphorylation and failed to differentiate into myofibroblasts by epithelial–mesenchymal transition [65]. In addition, data showing that cardiac myofibroblasts isolated from the infarction area express different VEGF isoforms including VEGF-A and receptor subtypes, may add support to the role of VEGF/VEGFR pathway in the modulation of the functionality of these cells in an autocrine and/or paracrine manner [101,102]. Coherently with our data, studies in mice demonstrated that VEGF-A delivery ameliorates tubulointerstitial fibrosis in unilateral ureteral obstruction model [103] and reduced fibrotic tissue within ischemic skeletal muscle tissue [89]. Moreover, the suppression of a number of profibrotic mechanisms related to myofibroblast activation was observed in lung-specific overexpressing VEGF-A transgenic mice treated with bleomycin [104], as an exacerbation was observed of bleomycin-induced fibrosis, which was associated with a massive increase of myofibroblasts, following the loss of myeloid cell-released VEGF-A in the damaged lung [105]. Of note, some studies have documented a reduction of the expression levels of VEGF-A and its receptors, including VEGFR-1, in lung tissue samples from idiopathic pulmonary fibrosis (IPF) patients associated with a progressive IPF phenotype [104,106].

## 5. Conclusions

In conclusion, our study provides new insights regarding the cellular and molecular mechanisms by which PRP may exert an antifibrotic action, demonstrating that it prevents fibroblast-myofibroblast transition via VEGF-A/VEGFR-1-mediated inhibition of TGF-β1/Smad3 signaling.

The main limitations of this study rely on the in vitro experimentation on cell lines and on the lack of a full characterization of the releasing profiles of the growth factors present in PRP that should be relevant to achieve a therapeutic translation of this approach. Moreover, experiments aimed to assess the effects of PRP on differentiated myofibroblasts, by evaluating the capability of this compound to modulate their fate—for example by inducing myofibroblast reversion to an inactive phenotype or senescence or apoptosis—should be of interest to support the antifibrotic action of PRP. However, despite these aspects, our study provides an experimental background for considering PRP as a potential therapeutic tool for those diseases where fibrosis plays a major etiological role.

## Supplementary Materials

The following are available online at http://www.mdpi.com/2073-4409/7/9/142/s1, **Figure S1.** Original western blot of α-sma and α-tubulin expression of Figure 1g. M: marker (Protein Marker VI (10–245) prestained, PanReac Applichem, VWR, Milan, Italy). **Figure S2.** Original western blot of VEGFR-1 and α-tubulin expression of Figure 4a. M: marker (Protein Marker VI (10–245) prestained, PanReac Applichem). **Figure S3.** Original western blot of α-sma and α-tubulin expression of Figure 6a. M: marker (Protein Marker VI (10–245) prestained, PanReac Applichem). **Figure S4.** Original western blot of Smad3 and β-actin expression of Figure 7a. M: marker (Protein Marker VI (10–245) prestained, PanReac Applichem). **Figure S5.** Original western blot of Smad3 and β-actin expression of Figure 7b. M: marker (Protein Marker VI (10–245) prestained, PanReac Applichem).
