# Supplementary material for: Platelet-Rich Plasma Prevents In Vitro Transforming Growth Factor-β1-Induced Fibroblast to Myofibroblast Transition: Involvement of Vascular Endothelial Growth Factor (VEGF)-A/VEGF Receptor-1-Mediated Signaling
Source: Cells. 2018 Sep 19;7(9):142. doi: 10.3390/cells7090142 (PMC6162453; doi:10.3390/cells7090142)
Supplement: Supplementary file 1 [file cells-07-00142-s001.pdf]

Supplementary Materials

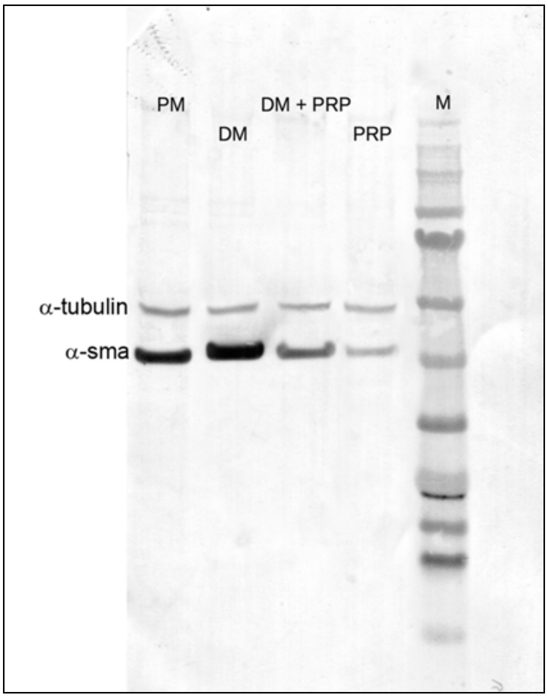

Figure S1. Original western blot of α-sma and α-tubulin expression of Figure 1 g. M: marker (Protein Marker VI (10 - 245) prestained, PanReac Applichem, VWR, Milan, Italy).

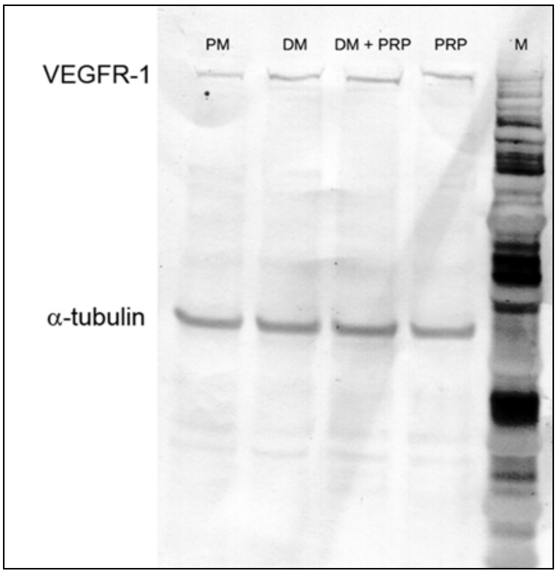

Figure S2. Original western blot of VEGFR-1 and α-tubulin expression of Figure 4a. M: marker **(**Protein Marker VI (10 - 245) prestained, PanReac Applichem).

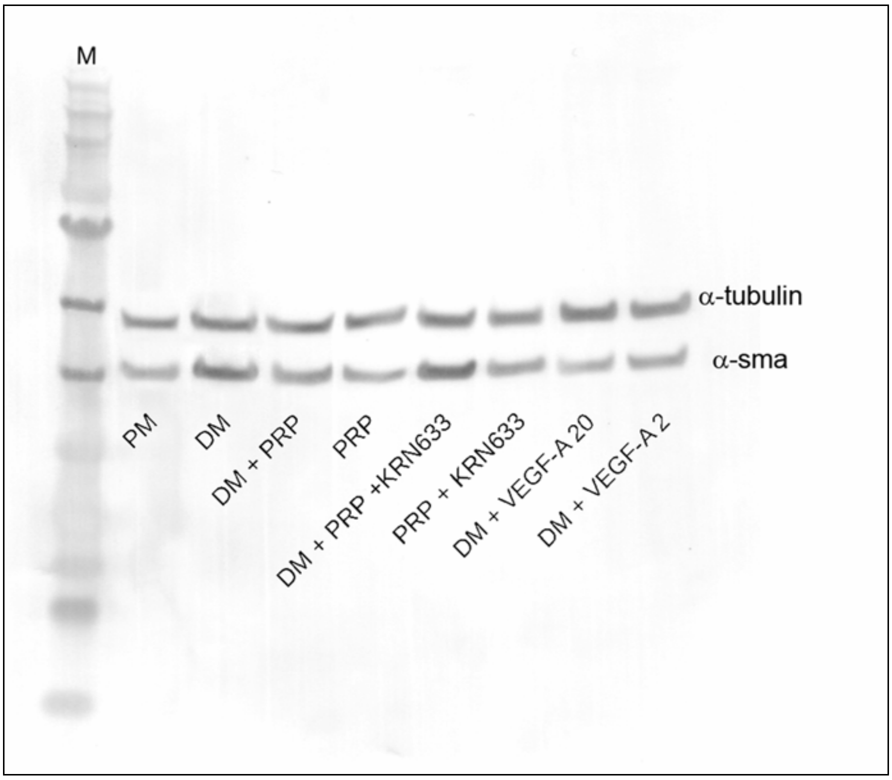

Figure S3. Original western blot of α-sma and α-tubulin expression of Figure 6a. M: marker **(**Protein Marker VI (10 - 245) prestained, PanReac Applichem).

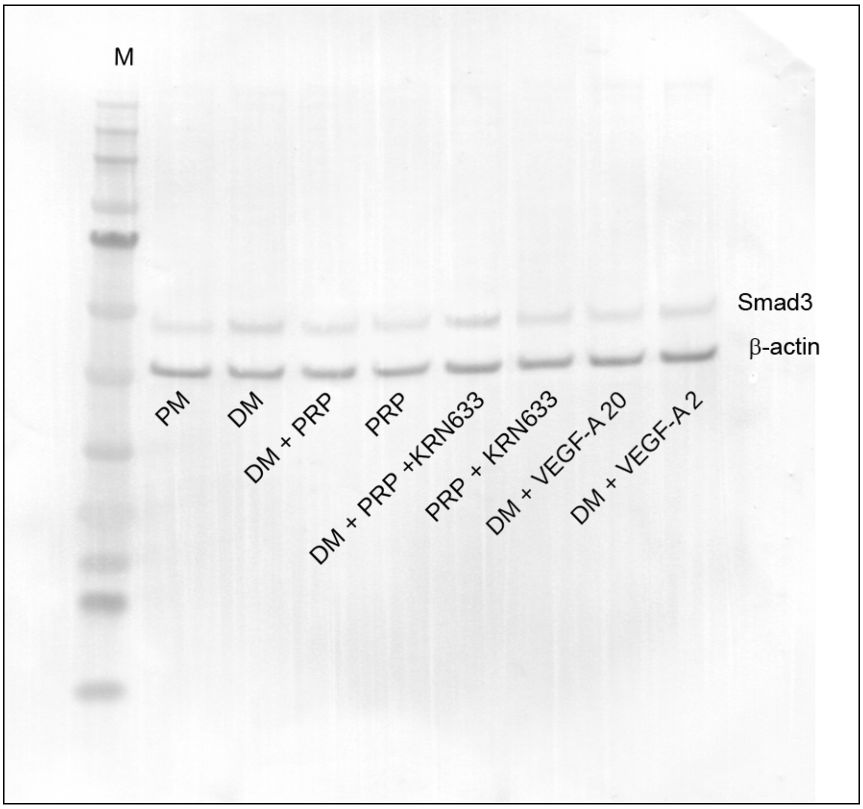

Figure S4. Original western blot of Smad3 and β-actin expression of Figure 7a. M: marker **(**Protein Marker VI (10 - 245) prestained, PanReac Applichem).

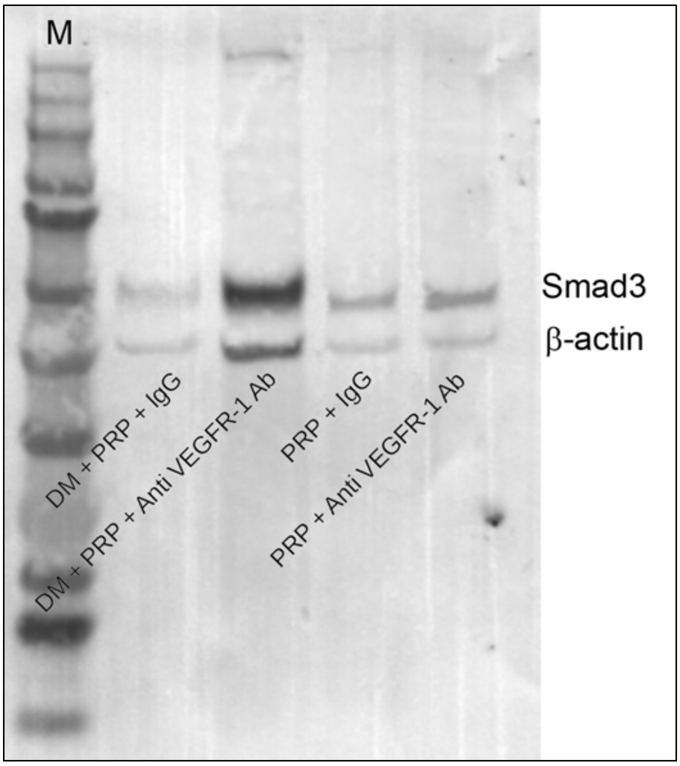

Figure S5. Original western blot of Smad3 and β-actin expression of Figure 7b. M: marker **(**Protein Marker VI (10 - 245) prestained, PanReac Applichem).
